# Precision Glycoproteomics Reveals Distinctive *N*-Glycosylation in Human Spermatozoa

**DOI:** 10.1016/j.mcpro.2022.100214

**Published:** 2022-02-18

**Authors:** Miaomiao Xin, Shanshan You, Yintai Xu, Wenhao Shi, Bojing Zhu, Jiechen Shen, Jingyu Wu, Cheng Li, Zexuan Chen, Yuanjie Su, Juanzi Shi, Shisheng Sun

**Affiliations:** 1 College of Life Science, Northwest University, Xi’an, Shaanxi Province, China; 2 Faculty of Fisheries and Protection of Waters, University of South Bohemia in Ceske Budejovice, South Bohemian Research Center of Aquaculture and Biodiversity of Hydrocenoses, Research Institute of Fish Culture and Hydrobiology, Vodnany, Czech Republic; 3 The Assisted Reproduction Center, Northwest Women and Children’s Hospital, Xi’an, China; 4 School of Computer Science and Technology, Xidian University, Xi’an, China

**Keywords:** spermatozoa, glycoproteomics, intact glycopeptides, glycan structures, mass spectrometry, ACN, acetonitrile, BP, biological processes, CC, cellular component, DAVID, database for annotation, visualization, and integrated discovery, DTT, dithiothreitol, FA, formic acid, FDR, false discovery rate, GO, gene ontology, IGP, intact glycopeptide, KEGG, Kyoto Encyclopedia of Genes and Genomes, MF, molecular function, RPLC, reverse-phase liquid chromatography, RT, room temperature, TFA, trifluoroacetic acid

## Abstract

Spermatozoon represents a very special cell type in human body, and glycosylation plays essential roles in its whole life including spermatogenesis, maturation, capacitation, sperm–egg recognition, and fertilization. In this study, by mapping the most comprehensive *N*-glycoproteome of human spermatozoa using our recently developed site-specific glycoproteomic approaches, we show that spermatozoa contain a number of distinctive glycoproteins, which are mainly involved in spermatogenesis, acrosome reaction and sperm:oocyte membrane binding, and fertilization. Heavy fucosylation is observed on 14 glycoproteins mostly located at extracellular and cell surface regions in spermatozoa but not in other tissues. Sialylation and Lewis epitopes are enriched in the biological process of immune response in spermatozoa, while bisected core structures and LacdiNAc structures are highly expressed in acrosome. These data deepen our knowledge about glycosylation in spermatozoa and lay the foundation for functional study of glycosylation and glycan structures in male infertility.

## Highlights

• A precision site-specific glycoproteome is documented in human spermatozoa.
• Distinctive glycoproteins and heavy fucosylation are detected in spermatozoa.
• Sialylation and Lewis epitopes are related to immune response of spermatozoa.
• Bisected core structures and LacdiNAc are enriched on acrosome of spermatozoa.

## Graphical Abstract

**Figure:**
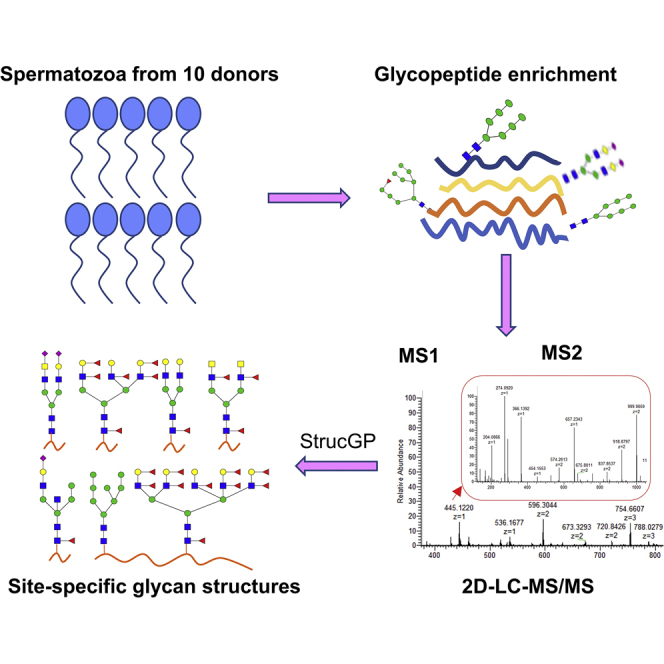

## In Brief

An in-depth *N*-glycoproteome map of human spermatozoa has been established by interpreting precise glycan structures at each glycosite, which allowed revealing a number of distinctive glycoproteins and heavily fucosylated glycans in spermatozoa. Sialylation and Lewis epitopes were enriched in the biological process of immune response, and highly expressed bisected core structure and LacdiNAc were detected in spermatozoa acrosome. These findings lay the foundation for subsequent functional studies of glycosylation and glycan structures in spermatozoa and male reproduction diseases.

Glycosylation is an important co- and posttranslational modification of proteins, playing fundamental roles in various biological processes (BP) and cellular activities, such as adhesion, interactions, structural and functional regulation (1, 2). Glycosylation can be further sorted into *N*-glycosylation (normally N-X-S/T and rarely N-X-C/V, X≠P) (3, 4), *O*-glycosylation (Ser/Thr residues via *N*-acetylgalactosamine), and rarely some other types (5, 6). Currently, *N*-glycosylation raises widespread concerns due to its huge potentials for current therapeutic targets and clinical biomarkers (7). While the complexity of glycosylation heterogeneity poses great challenges for comprehensive glycoproteome analysis.

Over the past decades, considerable progress has been made in the large-scale characterization of glycosylation events (8). Glycomic and deglycosylated glycoproteomic studies characterized liberated glycans or glycopeptides, which unfortunately lost information of glycosites or attached glycans (9, 10, 11). With the demand of knowing detailed information between glycosites and their corresponding glycans, intact glycopeptide (IGP) approaches have been developed to identify glycoproteins with site-specific glycan information in biological samples (5, 12, 13). However, most developed IGP analysis approaches assign compositions from the glycan database, which neither identify functional glycan structures nor distinguish different isomeric structures (13, 14, 15). Recently, a new IGP analysis software termed StrucGP developed by our team has been put forward to interpret precise glycan structures and identify new/rare glycans, as well as distinguish glycan isomeric glycoforms without relying on glycan database (16). This approach provides valuable insights into the interdependence of glycan structures and glycoprotein function.

Spermatozoon carries the male genetic information and works to complete its mission for successful fertilization (17). Glycosylation has been reported to accompany the whole process of gene transportation during accomplishment of fertilization task (18), such as spermatogenesis, maturation (19), capacitation (20, 21), sperm–egg recognition, and fertilization (22, 23). Spermatozoa surface possess glycoconjugates and glycocalyx for functioning in immune response, capacitation, and subsequent fertilization (24). Current glycomic and glycoproteomic studies on human spermatozoa have performed on either liberated glycan compositions or deglycopeptides, which cannot obtain the corresponding information between glycan structures and glycopeptides (25, 26). Consequently, the functional interactions of glycan structures and glycoproteins keep largely mysterious in human spermatozoa.

In this study, we applied our recently developed site-specific glycoproteomic method combined with 2D-LC-MS/MS analysis for in-depth characterization of glycan structures at each glycosite of glycoproteins in human spermatozoa. These sperm glycoproteomic data were compared with previously published glycoproteome and glycome data to determine the glycoproteins and glycan structures distinctively expressed in spermatozoa. Finally, the association of glycan substructures with different BP and cellular localizations was systematically explored to understand the structural function of glycans and glycoproteins in human spermatozoa.

## Experimental Procedures

### Experimental Design and Statistical Rationale

**Fig. 1:**
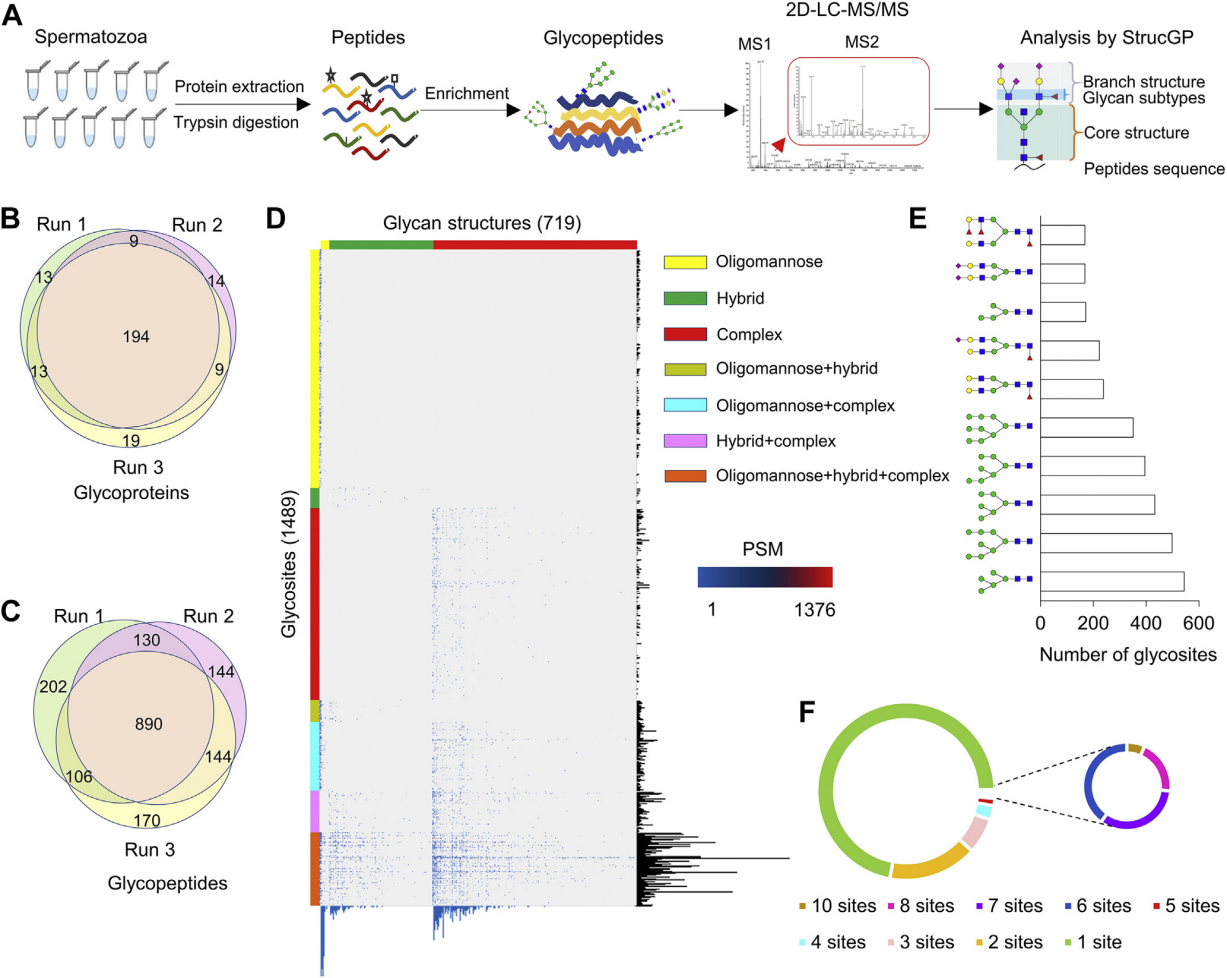
**In-depth glycoproteome characteristics of human spermatozoa.***A*, glycoproteomic workflow of human spermatozoa. Proteins were extracted from ten samples individually, and IGPs were enriched by hydrophilic cotton columns. Then IGPs from each sample were pooled for separation into 12 fractions *via* high-pH RPLC, and subjected to triplicate LC-MS/MS analyses per fraction. The IGPs were identified by StrucGP. *B* and *C*, overlaps of glycoproteins (*B*), glycopeptides (*C*) identified from triplicate LC-MS/MS data of a single fraction. *D*, heat map of identified IGPs based on glycan structures from human spermatozoa. The PSMs of IGPs, comprising of 719 N-glycans (*upper*) and 1489 glycosites (*left*), were exhibited in the heat map. The numbers of glycosites modified by each glycan and glycans at each glycosite were summarized at the *bottom* and *right parts* of the figure, respectively. The detailed information can be found in supplemental Table S3. *E*, top ten glycans detected in human spermatozoa based on the numbers of their modified glycosylation sites. *F*, numbers of glycosites on spermatozoa glycoproteins in human. IGP, intact glycopeptide; RPLC, reverse-phase liquid chromatography.

Spermatozoa proteins from ten healthy human donors were extracted and digested by trypsin individually, and IGPs were then enriched using hydrophilic cotton columns. Ten glycopeptide samples were pooled into one sample with the equal amount prior to separation into 12 fractions *via* high-pH reverse-phase liquid chromatography (RPLC) and subsequently subjected to triplicate LC-MS/MS analyses per fraction (Fig. 1*A*). Hence, a total of 36 LC-MS raw data (12 fractions x 3 runs/fraction) were generated for IGP identification using StrucGP. The false discovery rate of less than 1% (FDR<1%) was required for both peptide and glycan portions of IGPs. Functional analysis of glycoproteins, including gene ontology (GO) enrichment, and KEGG and reactome pathway used *p* value less than 0.05 as the significance.

### Sample Collection

Human semen was collected from healthy donors with the age between 27 and 34 years old (n = 10), according to the World Health Organization 2010 recommendations (27), in the Northwest Women's and Children's Hospital, Shaanxi Province, China. This study was approved by the Ethics Committee of Northwest Women's and Children's Hospital and Northwest University and abided by the Declaration of Helsinki principles. The clinical parameters of semen from ten healthy donors used in the present study are given in supplemental Table S1. Semen after 30 min liquefaction was centrifuged at 2000*g*, 4 °C for 15 min to separate spermatozoa (bottom pellets) from seminal plasma (upper supernatant). Spermatozoa were washed three times in phosphate-buffered saline (PBS, pH 7.4).

### Protein Digestion and Desalination

The collected human spermatozoa samples were denatured individually in 8 M urea/1 M NH_4_HCO_3_ and went through ultrasonication on ice by Ultrasonic Cell Distribution System. Thereafter, denatured samples were centrifuged at 15,000*g* for 20 min, and supernatant was collected for protein concentration measurement by BCA reagent (Beyotime). Spermatozoa proteins (1 mg) were reduced by 5 mM dithiothreitol (DTT) at 37 °C for 1 h with gentle shaking, then alkylated by 15 mM iodoacetamide at room temperature (RT) for 30 min in the dark. Subsequently, another 2.5 mM DTT was added and incubated for 10 min at RT. Protein samples were diluted twofold with deionized water and digested by sequencing grade trypsin (protein: enzyme, 100:1, w/w; Promega) at 37 °C for 2 h with gentle shaking as the first digestion. Afterward, samples were diluted fourfold with deionized water and sequencing grade trypsin (protein: enzyme, 100:1, w/w; Promega) was used to digest proteins into peptides again by incubation at 37 °C with gentle shaking overnight, namely the second digestion. The samples were acidified with trifluoroacetic acid (TFA) and centrifuged at 15,000*g* for 15 min to remove any particulate matter. The digested peptides were desalted with C18 column (Waters) and eluted with 50% acetonitrile (ACN)/0.1% TFA. The peptide concentration was measured by BCA reagent (Beyotime).

### Enrichment of N-Linked Intact Glycopeptides

The desalted peptides mixtures eluted from C18 column were adjusted to a final solvent composition of 80% ACN/1%TFA. Three microgram cotton was pushed into a 200 μl pipet tip as *in house* cotton column. Cotton columns were washed ten times by ddH_2_O and sequentially conditioned ten times by 80% ACN/1%TFA *via* aspirating. Samples in 80% ACN/1%TFA were loaded onto cotton columns 20 to 30 times and washed ten times by 80% ACN/1%TFA. The IGPs bound to cotton column were eventually eluted in 0.1% formic acid (FA). The concentrations of enriched IGPs were estimated by Ultra Trace Ultraviolet Spectrophotometer (Denovix). Afterward, equal amount of enriched IGPs (20 μg) from different individuals were pooled and dried by vacuum concentration with an RVC 2-18 CDplus concentrator (Christ).

### High-Performance Liquid Chromatography Fractionation

The dried IGPs were resuspended in 2% ACN/20 mM NH_4_COOH and fractionated through high pH reverse-phase high-performance liquid chromatography with Agilent RP Zorbax 300 Å Extend C18 column (250 mm × 4.6 mm, OD 5 μm) in an Agilent 1260 LC instrument. Intact glycopeptides were first separated into 96 fractions through a gradient of mobile phases A (2% ACN/5 mM NH_4_COOH, pH 10) and B (90% ACN/5 mM NH_4_COOH, pH 10) at a flow rate of 0.2 ml/min. A total of 120 min was used for gradient elution, 0 to 2% B for 7 min, 2 to 8% B for 35 min, 8 to 16% B for 35 min, 16 to 35% B for 10 min, 35 to 95% B for 5 min, 95 to 95% B for 28 min. Then, IGPs were integrated into 12 fractions and dried by vacuum centrifuging.

### LC-MS/MS Analysis

Intact glycopeptides in each fraction underwent three LC-MS/MS runs on an Orbitrap Fusion Lumos mass spectrometer (Thermo Fisher Scientific). About 1 μg IGPs were separated by an Easy-nLC 1200 system with a 75 μm × 50 cm Acclaim PepMap-100 C18 analytical column protected by a 75 μm × 2 cm trapping column. The mobile phase flow rate was 0.2 μl/min and consisted of 0.1% FA in ddH_2_O (A) and 0.1% FA/80% ACN (B). A complete run of 240 min was set as follows: 3 to 9% B for 88 min, 9 to 31% B for 120 min, 31 to 40% B for 15 min, 40 to 99% B for 4 min, 99% B for 13 min. MS analysis was performed using Orbitrap Fusion Lumos mass spectrometer (Thermo Fisher Scientific). The spray voltage (+) was set at 2300V. Orbitrap spectra (AGC 4 × 10^5^) were collected from 375 to 2000 m/z at a resolution of 120K followed by data-dependent HCD-MS/MS (AGC 2 × 10^5^, collected from 120 to 3000 m/z at a resolution of 30K, collision energy was 20% and 33%, respectively). An isolation window of 2.0 M/Z was used. Charge state screening enabled to reject unassigned and singly charged ions. A dynamic exclusion time of 20 s was set for each precursor ion after being selected once.

### Intact Glycopeptide Identification

The identification of IGPs was performed by our newly developed software, StrucGP 1.0 (16). Briefly, all “Raw” format MS data were first converted to “mzML” format by Trans-Proteomic Pipeline (TPP, v5.0.0) (28). IGPs analyses were performed by StrucGP using the built-in glycan branch structure database from StrucGP 1.0 (16) and the UniProtKB of human protein databases (20,341 entries, downloaded from http://www.uniprot.org May 2020). The protein enzymatic digestion was set as trypsin with max two missed cleavage sites and potential glycosite-containing peptides were screened with the N-X-S/T motif (X is any amino acid except Proline). The carbamidomethylation (C, +57.0215 Da) was as a fixed modification, and oxidization (M, +15.9949 Da) as a dynamic modification. The mass tolerances for MS1 and MS2 were set at 10 ppm and 20 ppm, respectively. For the Y ions determination, an optional mass shift of ±1 Da or ±2 Da was allowed in addition to the 20 ppm mass tolerance in MS2. Finally, FDR evaluation of peptides and glycans (FDR < 1%) was determined by a decoy database and a decoy spectrum, respectively (16).

### Bioinformatics Analysis

GO enrichment analysis was performed by the database for annotation, visualization, and integrated discovery (DAVID) (https://david.ncifcrf.gov/) to identify biological themes, including BP, cellular component (CC), and molecular function (MF) (29). Kyoto Encyclopedia of Genes and Genomes (KEGG) and reactome pathway analysis was performed by using ClueGO plug-in and Cluepedia of Cytoscape software (30). These pathway enrichment analyses were performed to search for the associated important pathway information and key glycoproteins (after correcting for multiple term testing using the two-sided hypergeometric test and procedure of Bonferroni−Hochberg). *p* value less than 0.05 was regarded as the significant pathway.

## Results

### In-Depth Glycoproteome Characteristics of Human Spermatozoa

With well-established site-specific glycoproteomic approaches coupled with 2D-LC-MS/MS (Fig. 1*A*), a total of 10,355 unique N-linked IGPs from 968 glycoproteins, consisting of 1489 glycosites and 719 glycans with distinct structures (292 glycan compositions) were screened out in human spermatozoa (supplemental Fig. S1 and supplemental Table S2), which is the most comprehensive glycoproteome map of human spermatozoa till now. Over 95% of glycoproteins (Fig. 1*B*) and 87% of glycopeptides (Fig. 1*C*) were overlapped among triplicate LC-MS analyses of a single fraction, indicating a reasonably high reproducibility of the mass spectrometry data. Based on the heat map of site-specific *N*-glycans from spermatozoa (Fig. 1*D* and supplemental Table S3), the majority of glycosites were occupied by high mannose (36.3% of PSMs) and complex glycans (29.2%), followed by a combination of three different types of glycans (11.5%). The top ten glycans appearing at different glycosites (based on PSMs) were mostly high mannose (N2H5, N2H8, N2H6, N2H7, N2H9, N2H4) and diantennary glycans (N4H5F1, N4H5F1S1, N4H5S2, N4H5F2, N4H5F3) (Fig. 1*E*). The majority of glycoproteins (71.8%) contained one glycosite (Fig. 1*F*), while other 273 glycoproteins (28.2%) contained two to ten glycosites.

### Precise *N*-Glycan Structure Interpretation in Human Spermatozoa

**Fig. 2:**
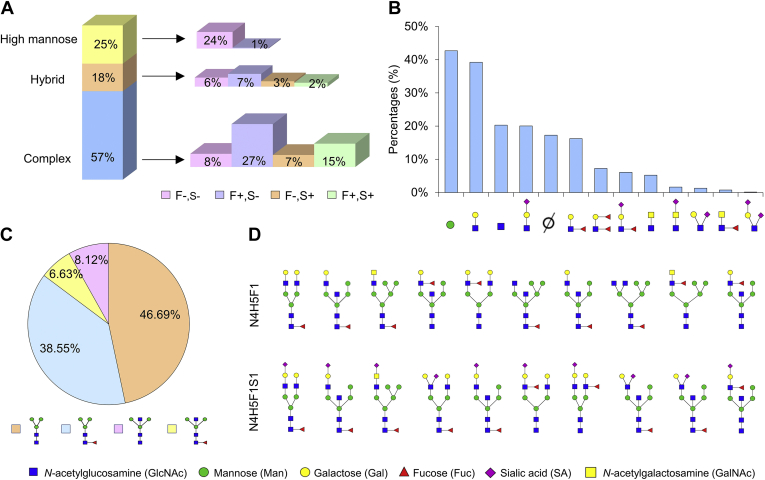
**Precise *N*-glycan structure interpretation in human spermatozoa.***A*, abundance of different types of glycans with F and S distribution. F: fucose, S: sialic acid, “−”: excluding, “+”: including. *B* and *C*, distribution of core (*B*), branch structures (*C*) on IGPs in human spermatozoa. The percentages were calculated based on the unique glycopeptides modified by each core or branch glycan structure. “∅”: lack of one branch. *D*, glycan isoforms identified in human spermatozoa. 10 glycan isoforms were identified with glycan compositions of both N4H5F1 and N4H5F1S1. N: HexNAc; H: hexose; F: fucose; S: sialic acid.

As described above, a total of 719 precise glycan structures have been identified in human spermatozoa (supplemental Fig. S1). These glycans included high mannose, bi-, tri-, and tetra-antennary bisecting types, and bi-, tri-, and tetra-antennary core-fucosylated oligosaccharides terminated with Lewis structures (including Lewis^x/a/y/b^), as well as bi-, tri-, and tetraantennary core-fucosylated complex type *N*-glycans with antennae capped with sialic acid. Among the glycans on unique IGPs, 57.31% were complex glycans, followed by hybrid (17.87%) and oligomannose glycans (24.83%) (Fig. 2*A*).

These spermatozoa *N*-glycans comprised four types of core structures and 13 types of branch structures. Among 13 distinct branch structures, oligo-mannose (labeled as Hex) and LacNAc (HexNAc+Hex) accounted for 42.69% and 39.22% of unique IGPs, respectively. Sole HexNAc and HexNAc+Hex+Neu5Ac took up 20.30% and 20.04% of IGPs (Fig. 2*B*). Lewis^y/b^, sialylated Lewis^x/a^, and other remaining structures existed merely at relatively low abundances (Fig. 2*B*). Referring to four types of core structures, the common core structure (HexNAc_2_Hex_3_, 46.69%) and fucosylated core structure (38.55%) accounted for the predominance of all unique IGPs, while the bisected core structure with and without core fucosylation only occupied 6.63% and 8.12%, respectively (Fig. 2*C*).

Since 719 *N*-glycan structures were sorted into 292 different compositions in present study, lots of glycan compositions actually consisted of different isoforms (supplemental Table S4). Indeed, our data showed that up to ten distinct glycan structure isoforms can be discriminated from one single composition, such as N4H5F1S1 and N4H5F1 (Fig. 2*D*). Most glycans with isomers were hybrid and complex glycans. These glycan isoforms attached at the same peptide could be identified at the MS/MS level, like different isomers of the N4H5F1 glycan composition distinguished by feature B and Y ions in the MS/MS spectra (supplemental Fig. S2).

### Distinctive Functional Glycoproteins Identified in Human Spermatozoa

**Fig. 3:**
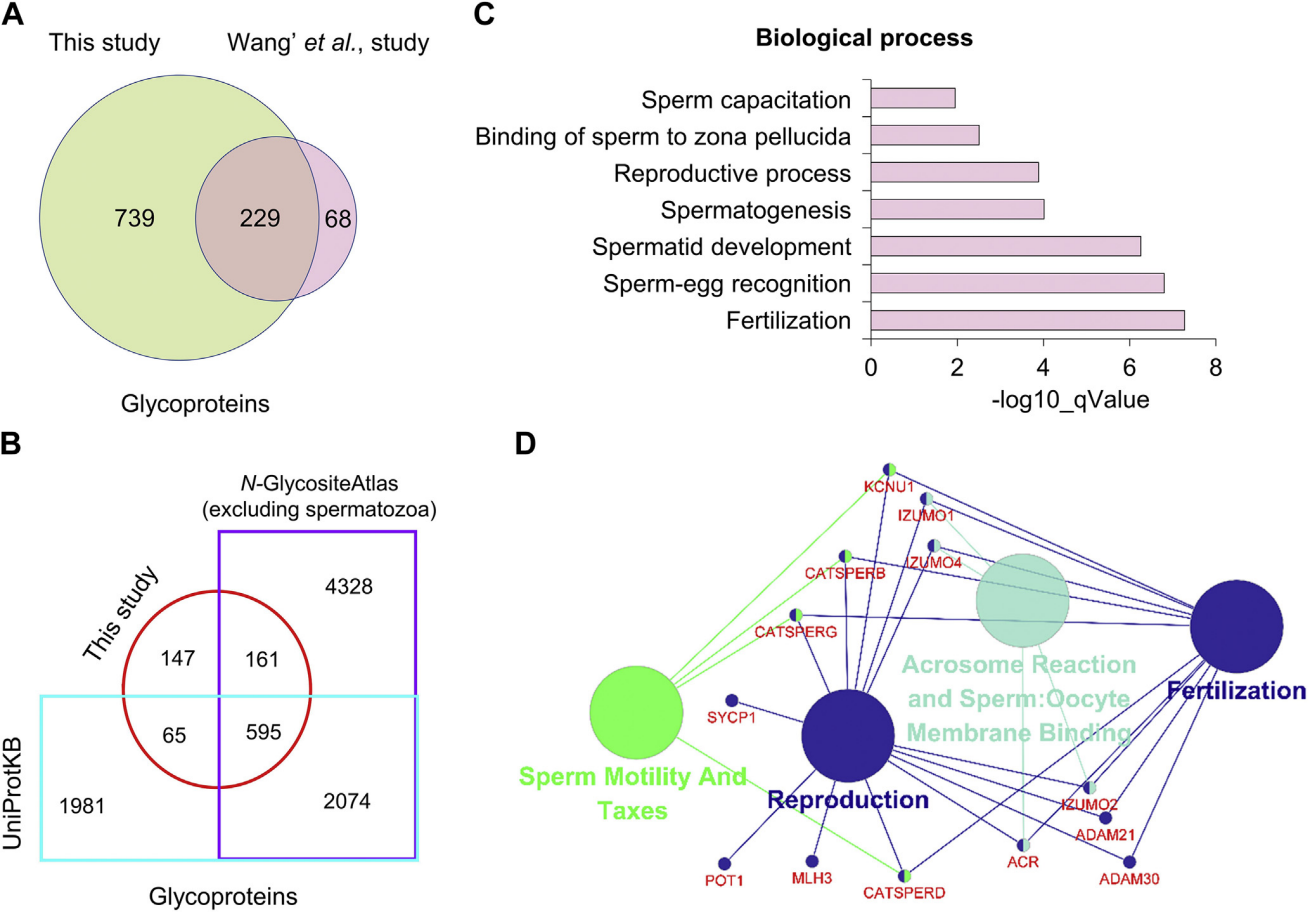
**Distinctive functional glycoproteins identified in human spermatozoa.***A* and *B*, spermatozoa glycoproteins identified currently comparing with the previous study (25) (*A*), *N*-glycositeAtlas (31) and UniProtKB (*B*). *C* and *D*, biological processes (*C*) and reactome pathways (*D*) involved by the glycoproteins distinctively identified in current study.

Spermatozoa glycoproteins identified in this study were compared with previously published spermatozoa glycoproteome data, and glycoproteins in two N-linked glycoprotein databases—UniProtKB (http://www.uniprot.org) and *N*-glycositeAtlas (31). In current study, 968 identified glycoproteins covered 229 of 297 human spermatozoa glycoproteins published previously (25), and other 739 glycoproteins were characterized newly in human spermatozoa (Fig. 3*A*). Moreover, 660 glycoproteins coincided with known human glycoproteins in UniProtKB database, while other 308 glycoproteins had no reports in the database (Fig. 3*B*). Importantly, 212 glycoproteins were uniquely identified in spermatozoa, which have never been detected in other human tissues or cells using mass-spectrometry-based glycoproteomic approaches according to the *N*-GlycositeAtlas database (31) (Fig. 3*B* and supplemental Table S5). In-depth bioinformatic analysis of these 212 glycoproteins demonstrated that these proteins mainly participated in very specific BP of spermatozoa, such as binding of sperm to zona pellucida, reproductive process, spermatogenesis, spermatid development, sperm–egg recognition, fertilization (Fig. 3*C*). Similarly, based on the reactome pathway analysis, these glycoproteins were mainly involved in fertilization, reproduction, acrosome reaction, and sperm:oocyte membrane binding, as well as sperm motility and taxes (Fig. 3*D*).

### Heavy Fucosylation in Human Spermatozoa

**Fig. 4:**
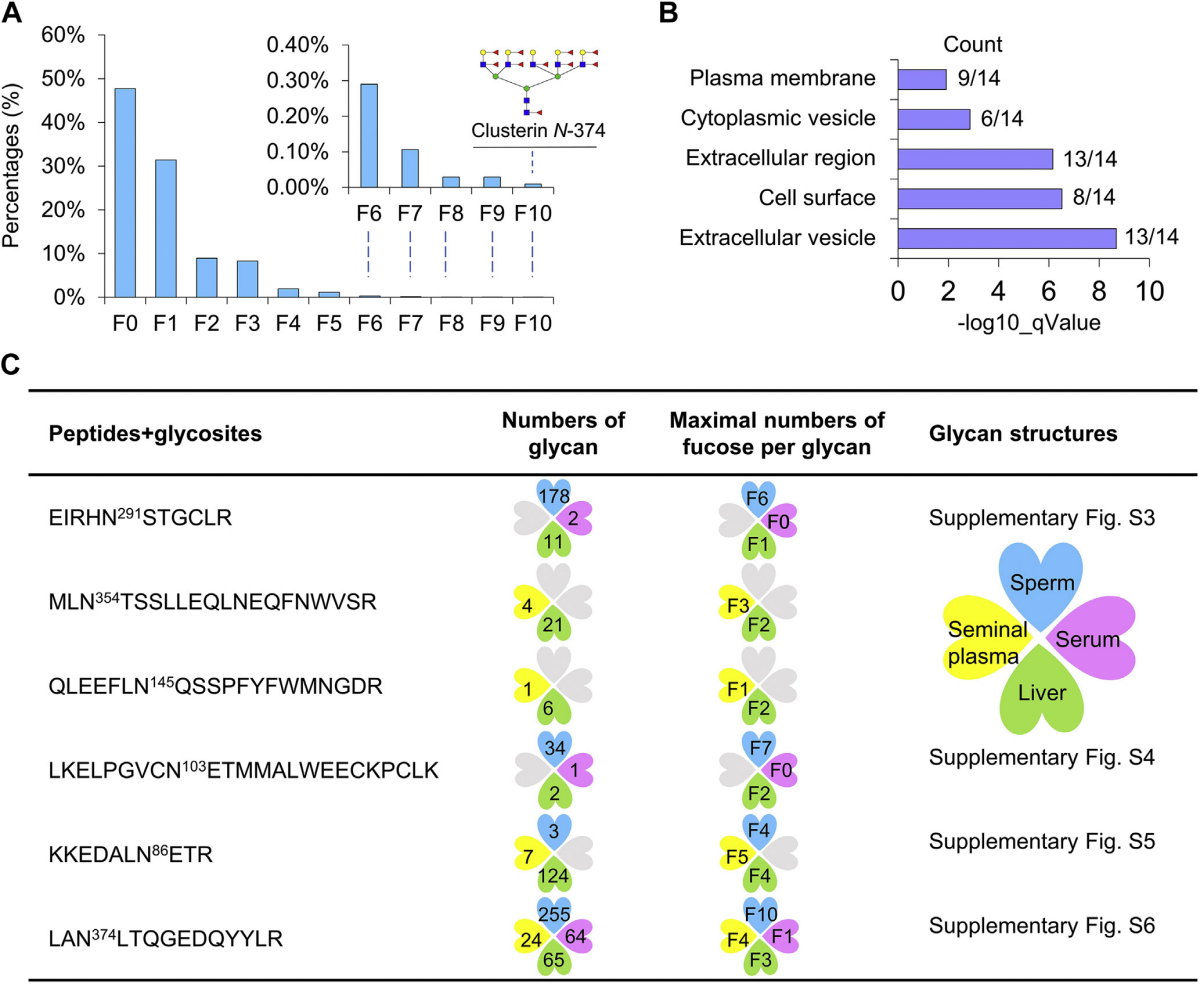
**Heavy fucosylation in human spermatozoa**. *A*, proportion of different numbers of fucoses on IGPs in spermatozoa. *B*, cell components of glycoproteins modified with at least six fucoses per glycan. *C*, glycopeptides and glycans on clusterin (with up to ten fucoses) identified in the spermatozoa, seminal plasma (35), liver (33), and serum (34). F: fucose. IGP, intact glycopeptide.

Heavy fucosylation was discovered in human spermatozoa. There were 52% of unique IGPs fucosylated in human spermatozoa (Fig. 4*A*), and 14 glycoproteins were modified by glycans with at least six fucoses per glycan (supplemental Table S6). Remarkably, up to ten fucoses per glycan were detected on glycosite *N*-374 of clusterin (Fig. 4*A*). Subsequent cellular component of GO enrichment revealed most glycoproteins with heavy fucosylation (above six fucoses per glycan) localizing on extracellular and cell surface regions (Fig. 4*B*). As a case study, four *N*-glycopeptides from clusterin, a glycoprotein that was mainly involved in sperm capacitation and immune tolerance in the female reproductive tract (32), were modified by heavy fucosylation (up to ten fucose per glycan) in human spermatozoa. The majority of glycans attached at all four *N*-glycosylation sites of clusterin were complex and hybrid glycans with Lewis^x/a^ (30%), Lewis^y/b^ (21%), and sialylated Lewis^x/a^ (19%). The maximal fucoses per glycan and glycan structures on each glycosite of clusterin were displayed (supplemental Figs. S3–S6, and supplemental Table S7). Especially, the interpretation of glycan structures N7H8F10 on peptide：LANLTQGEDQYYLR of clusterin by spectrogram was drawn (supplemental Fig. S7). Intriguingly, no heavy fucosylation was identified on clusterin in other human tissues and body fluids. For example, maximal three fucoses per glycan were identified in the liver (33), and only one fucose per glycan was identified in serum (34) (Fig. 4*D*). Specially, maximal five fucoses were exhibited on intact glycopeptides of clusterin in seminal plasma (35) (Fig. 4*C*), although heavy fucosylation (up to nine) has also been identified in seminal plasma glycans without peptides information (36). These data indicated that heavy fucosylation might be a distinctive feature of spermatozoa.

### Enriched Sialylation and Lewis Epitopes in the BP of Immune Response

**Fig. 5:**
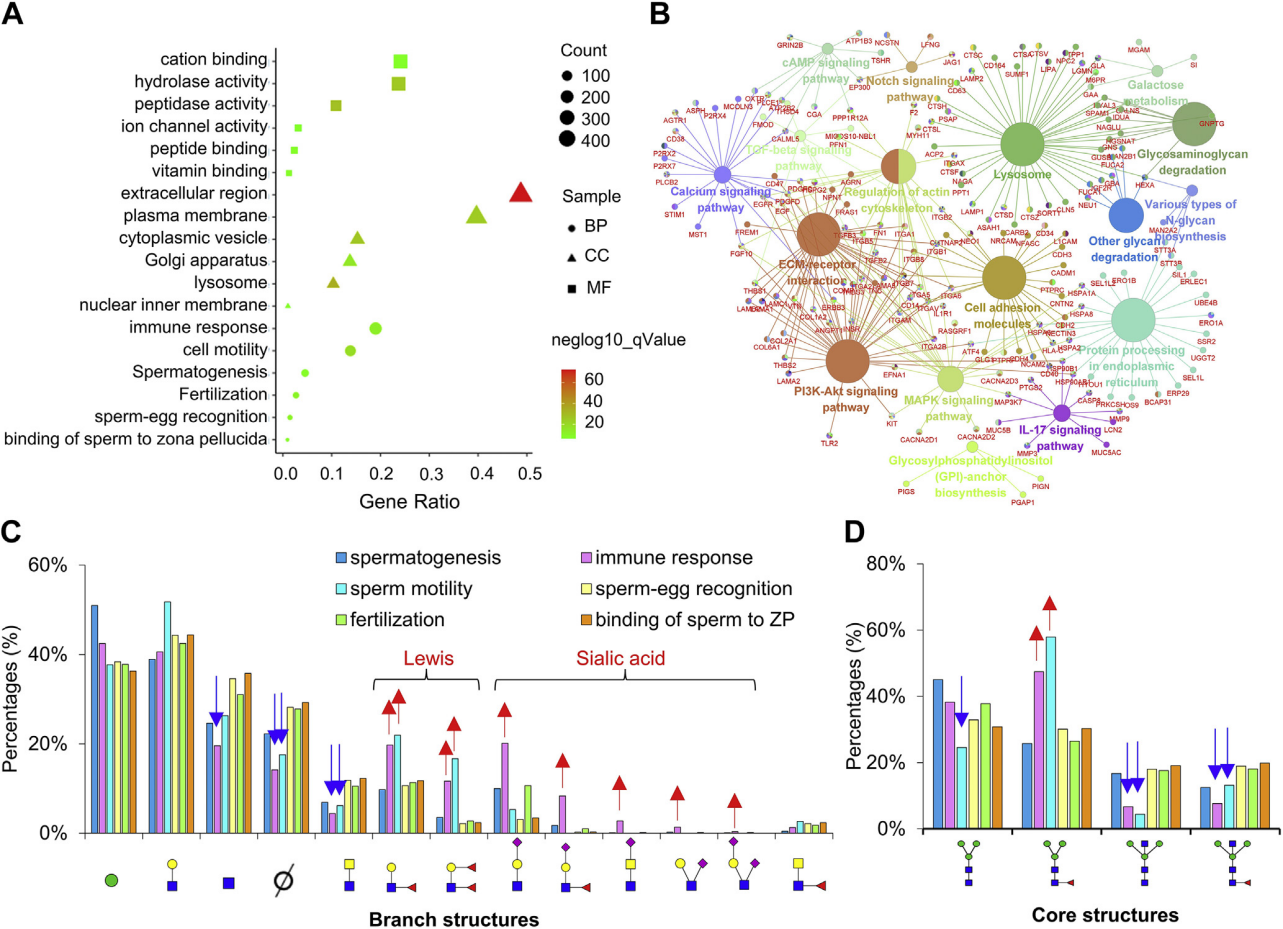
**Enrichment of sialylation and Lewis structures in the biological process(BP)of immune responseinspermatozoa**. *A*, molecular function (MF), cellular component (CC) and BP of total glycoproteins identified in human spermatozoa. *B*, KEGG pathway analysis of total glycoproteins in human spermatozoa. *C* and *D*, comparison of branch (*C*) and core (*D*) glycan structures on glycoproteins associated with different BP. “∅”: lack of one branch. KEGG, Kyoto Encyclopedia of Genes and Genomes.

A functional annotation of glycoproteins from human spermatozoa was performed using DAVID and divided into different categories: “BP,” “cellular components,” and “MFs” (Fig. 5*A*). Although there are different categories for each of the divisions made, the most important were those involved in immune response, cell motility, spermatogenesis, fertilization, sperm–egg recognition, binding of sperm to zona pellucida in case of BP (Fig. 5*A*); extracellular region, plasma membrane, cytoplasmic vesicle, Golgi apparatus, lysosome, nuclear inner membrane in case of cellular components; cation binding, hydrolase activity, peptidase activity, ion channel activity, peptide binding, and vitamin binding for MFs (Fig. 5*A*).

To predict the relevant molecular interaction, reaction, and relation networks of glycoproteins in human spermatozoa, the KEGG pathway analysis was conducted using ClueGO plug-in from Cytoscape software. The most important pathways enriched by spermatozoa glycoproteins were glycosaminoglycan degradation, calcium signaling pathway, protein processing in endoplasmic reticulum, lysosome, notch signaling pathway, cell adhesion molecules, other glycan degradation, glycosylphosphatidylinositol-anchor biosynthesis, various types of *N*-glycan biosynthesis, IL-17 signaling pathway, MAPK signaling pathway, regulation of actin cytoskeleton, PI3K-Akt signaling pathway, ECM–receptor interaction, TGF-beta signaling pathway, galactose metabolism, cAMP signaling pathway (Fig. 5*B*).

To further explore the functional interactions between glycan structures and glycoproteins, we compared the core and branch glycan structures on glycoproteins from different BP subsequently. Branch structures with salic acid were expressed outstanding in the BP of immune response in comparison with other BP (Fig. 5*C*). Similarly, branch structures of Lewis structures were also displayed highly in the BP of immune response and sperm motility (Fig. 5*C*). Correspondingly, the fucosylated core structure was much higher on glycoproteins involved in immune response and sperm motility than other BP (Fig. 5*D*). The integrating information revealed that sialylation and Lewis structures may play special roles in the specific spermatozoa BP of immune response.

### Highly Expressed Bisecting *N*-Glycans on Acrosome

**Fig. 6:**
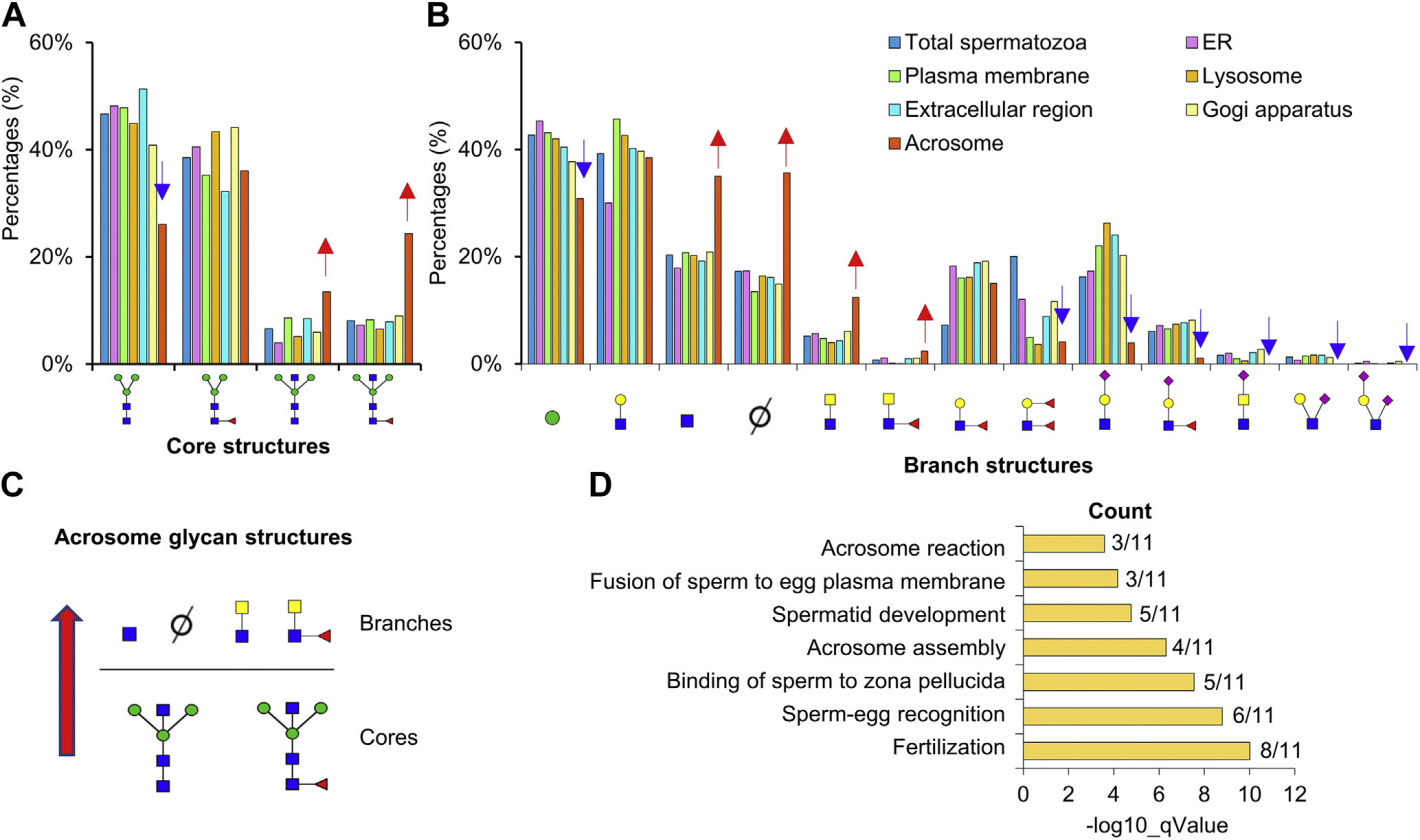
**High expression of bisecting N-Glycans on acrosome.***A* and *B*, core (*A*) and branch (*B*) structures on glycoproteins identified in different spermatozoa localizations. “∅”: lack of one branch. *C*, the core and branch structures differentially expressed in acrosome in comparison with other localizations of spermatozoa. *D*, biological processes of glycoproteins with bisecting glycan structures on spermatozoa acrosome.

According to different distributions of glycoproteins in human spermatozoa, we also compared the branch and core structures to gain insight into the demand of glycan structures in different cellular localization. Intriguingly, bisected core structure (with and without core fucosylation) was highly expressed in acrosome compared with the glycoproteins in other localization and total spermatozoa (Fig. 6*A* and supplemental Fig. S8). The presence of core bisecting *N*-glycans was known to simplify the branch structures by precluding further processing and elongation of *N*-glycans (37, 38). Within expectation, the complex branch structures such as Lewis and sialylation were significantly decreased on acrosome. Contrarily, simple branch structures of HexNAc and even the lack of one branch increased (Fig. 6, *B* and *C*). Besides, special branch structures of LacdiNAc (with and without fucosylation) were also increased in acrosome in comparison with total spermatozoa (Fig. 6, *B* and *C*). Interestingly, all 11 glycoproteins with LacdiNAc glycans in acrosome were also the glycoproteins with bisected core structures (supplemental Table S8), and these glycoproteins mainly participated in the BP of fertilization, sperm–egg recognition and fusion, acrosome reaction, *etc.* (Fig. 6*D*). All these data indicated that the LacdiNAc structures might work together with bisected core structures for special functions of acrosome.

## Discussion

As an important generative cell in males, spermatozoon owns many distinct features comparing with other types of human cells, likely progressive movement, survival in allogeneic immune response, and acrosome structure. These features are prerequisites for spermatozoa to accomplish their mission of fertilization. Genomics and proteomics studies have performed broadly on spermatozoa to understand the mechanism of spermatogenesis and further reproduction (39, 40, 41). In addition, glycomic and glycoproteomic studies have indicated the accompanying of glycosylation in the whole process of spermatogenesis, maturation, and fertilization (24). Nevertheless, these studies focused on either a small number of glycoproteins without glycan information or speculative glycan structures without corresponding glycosite (25, 26). This makes it difficult to further investigate the functions of *N*-glycosylation in depth, such as the mediation of glycan structures on the specific glycosites of glycoprotein function. In current study, we applied a site-specific glycoproteomic approach (16) to characterize glycoproteins with both glycosites and attached glycan structures precisely in human spermatozoa. Furthermore, 2D-LC-MS/MS ensured the landscape of glycoprotein identification even with low abundance. Based on the above strategies, a total of 10,355 IGPs with 719 glycan structures were identified from 968 spermatozoa glycoproteins, representing the largest and most comprehensive spermatozoa glycoproteome map till now. These achievements possessed distinct advantages in comparison with 554 *N*-glycosylation sites and 297 glycoproteins identified previously with loss of glycan information in human spermatozoa (25). Within our expectation, a portion of glycoproteins were uniquely identified in human spermatozoa, involving in the specific BP and pathways, likely spermatozoa development and fertilization. This further attested the comprehensiveness, depth, and reliability of glycoproteins identified in present study.

Heavy fucosylation has been detected in spermatozoa previously by using the glycomic approach (26), which unfortunately could not obtain the corresponding glycoproteins information. In this study, we are capable of detecting heavy fucosylation on specific glycoproteins precisely and further analyze their localizations and potential functions. It was indicated that glycoproteins modified by glycans with above six fucoses mostly localized on plasma membrane and extracellular region. Comparing with other tissues, such as human liver with maximal three fucoses per glycan, we realized the uniqueness of heavy fucosylation in human spermatozoa. Especially, we detected heavy fucosylation (up to ten fucoses) on clusterin in spermatozoa, which was in sharp contrast to few fucoses on clusterin in the serum (34) and liver (33), as well as in some mouse tissues based on IGPs (16). These findings indicated that *N*-glycans on clusterin might be tissue-specific. Since heavy fucosylation encompassed clusterin distinctively in human spermatozoa, the structural study of heavy fucosylation on clusterin function will be facilitated in human spermatozoa.

Since the corresponding glycan structure information has been obtained on glycoproteins of human spermatozoa, analysis of glycan structure regulation on glycoprotein function became possible during cellular activities and biological function (19). In present study, we discovered the high expression of sialylated branch structures and Lewis epitopes in the BP of immune response. Abundant sialylation was demonstrated to facilitate tolerance of female innate pattern recognition molecules when “foreign cells” spermatozoa passed through the onslaught of female immune factors for fertilization (20, 42). During this journey, appropriate sialylation acted as a mask for sperm antigens and protected spermatozoa from excessive phagocytosis by leucocytes (43). Lewis structures with core fucosylation contributed to block adaptive antigen directed against human sperm in both male and female reproductive systems (26). Coordinately, glycoproteins with heavy fucosylation (above six fucoses per glycan) were mainly localized on extracellular region and plasma membrane. This was consistent with complicated branch (Lewis structures), and core structures (fucosylated core structures) tend to be involved in the BP of immune response, which occurred in spermatozoa plasma membrane and extracellular region.

Different from the abundant common core structure and fucosylated core structure, bisecting *N*-glycans only occupied a minor portion of the total IGP profile in human spermatozoa. Surprisingly, significant highly expressed bisected core structures were detected on acrosome in comparison with total spermatozoa. Accordingly, the branch structures became shorter and rare LacdiNAc structures increased on acrosome as well. This agreed with the opinion that presence of bisected core structure prevented the formation of highly-branched species, such as the β1-6-GlcNAc (44, 45). Moreover, overexpression of bisected core structure was demonstrated to dramatically suppress α2-3-sialylation, but not α2-6-sialylation (38). Consistently, branch structures of all sialylated Lewis^x/a^, LacNAc, and LacdiNAc were significantly decreased, but not disappeared completely on acrosome glycoproteins. In addition, bisected core structures were demonstrated to be on sperm surface (26), likely enriched on acrosome in the present study. Based on all above analyses, highly expressed bisected core structures, along with simple and special branch structures on acrosome, may make a significant contribution in spermatozoa function.

It should be emphasized that the left/right branches of glycan structures were not actually distinguished using StrucGP v1.0 (16), and the structural interpretation of fucose-containing glycans might be affected by the rearrangement of the fucose residues in these glycans during mass-spectrometry-based fragmentation (46). In addition, the chemical modifications of *N*-glycans such as phosphorylation and sulfation, which might exist on sperm *N*-glycans (47, 48), were not considered and therefore could not be identified in this study. Also, additional experimental data are still required for further validation of the distinctive glycosylation and subglycan structures, as well as their biological functions in spermatozoa.

## Conclusion

Glycosylation participates in the whole process of spermatogenesis, maturation, capacitation, sperm–egg recognition, and fertilization. However, we are only beginning to truly understand what glycosylation looks like and how glycosylation functions in spermatozoa. In the present study, an in-depth glycoproteome map of human spermatozoa has been established by determining the precision glycan structures at each glycosylation site, which overcomes the drawbacks of glycomics and traditional glycoproteomics focusing on either released glycan compositions or deglycosylated peptides. The IGPs based on glycan structure interpretation allowed to characterize the distinctive glycosylation in spermatozoa systematically and at meticulous levels. These include many glycoproteins and heavily fucosylated glycans uniquely identified in the whole spermatozoa, as well as the special glycan characteristics in specific organisms or BP of spermatozoa. These achievements will be greatly beneficial for further in-depth functional investigation of glycan structures and glycoproteins in human spermatozoa, as well as the glycosylation regulation of sperm dysfunction and male reproductive diseases.

## Data Availability

The mass spectrometry data, and all spectra for identified glycopeptides in human spermatozoa, have been deposited to the ProteomeXchange Consortium (http://proteomecentral.proteomexchange.org) *via* the PRIDE partner repository (49) with the dataset identifier PXD026649.

## Supplemental data

This article contains supplemental data.

## Conflict of interest

The authors declare no competing interests.
