# Supplementary material for: Precision Glycoproteomics Reveals Distinctive N-Glycosylation in Human Spermatozoa
Source: Mol Cell Proteomics. 2022 Feb 18;21(4):100214. doi: 10.1016/j.mcpro.2022.100214 (PMC8958358; doi:10.1016/j.mcpro.2022.100214)
Supplement: Supplemental Figures S1–S8, Tables S1 and S4 [file mmc1.docx]

**Supplementary Information for:**

**Precision glycoproteomics reveals distinctive *N-*glycosylation in human spermatozoa**

Miaomiao Xin^1,2,#^, Shanshan You^1,#^, Yintai Xu^1,#^, Wenhao Shi^3^, Bojing Zhu^1^, Jiechen Shen^1^, Jingyu Wu^1^, Cheng Li^1^, Zexuan Chen^1^, Yuanjie Su^4^, Juanzi Shi^3,^*, Shisheng Sun^1,^*

*^1^College of Life Science, Northwest University, Xi’an, Shaanxi Province 710069, P. R. China,*

*^2^Faculty of Fisheries and Protection of Waters, University of South Bohemia in Ceske Budejovice, South Bohemian Research Center of Aquaculture and Biodiversity of Hydrocenoses, Research Institute of Fish Culture and Hydrobiology, Vodnany 38925，Czech Republic,*

*^3^The Assisted Reproduction Center, Northwest Women’s and Children’s Hospital, Xi’an, China,*

*^4^School of Computer Science and Technology, Xidian University, Xi’an, 710004, P. R. China*

^#^These authors contributed equally.

***Correspondence:**

Sun S: [suns@nwu.edu.cn](mailto:suns@nwu.edu.cn); Shi J: [shijuanzi@126.com](mailto:shijuanzi@126.com)

**Supplementary Material**

1. **Supplementary Figures**

**Figure S1.** Precise structures of 719 glycans identified in human spermatozoa.

**Figure S2** Spectra of glycopeptides with two glycan isoforms of N4H5F1 on the same peptide.

**Figure S3.** The glycan structures on peptide: EIRHNSTGCLR of clusterin in human spermatozoa.

**Figure S4.** The glycan structures on peptide: LKELPGVCNETMMALWEECKPCLK of clusterin in human spermatozoa.

**Figure S5.** The glycan structures on peptide: KKEDALNETR of clusterin in human spermatozoa.

**Figure S6.** The glycan structures on peptide: LANLTQGEDQYYLR of clusterin in human spermatozoa.

**Figure S7.** The annotations of glycan structures N7H8F10 on clusterin from de novo software based on MS/MS data.

**Figure S8** The glycan structures on acrosome of human spermatozoa.

1. **Supplementary Tables (Table S2, S3, S5, S6, S7 and S8 as separate files)**

**Table S1.** Clinical information of semen parameters used in this study.

**Table S2.** Intact glycopeptides identified in human spermatozoa by StrucGP.

**Table S3.** Heatmap of intact glycopeptides with actual glycosites and their corresponding glycans in human spermatozoa identified by StrucGP.

**Table S4.** Glycan compositions with different glycan isoforms identified in spermatozoa.

**Table S5.** Glycoproteins uniquely identified in spermatozoa.

**Table S6.** Glycoproteins modified by heavily fucosylated *N-*glycans in spermatozoa (at least six fucoses per glycan).

**Table S7.** Glycan structures on four peptides of clusterin in human spermatozoa.

**Table S8.** Intact glycopeptides and glycoproteins identified on acrosome of human spermatozoa.

**1. Supplementary Figures**

**
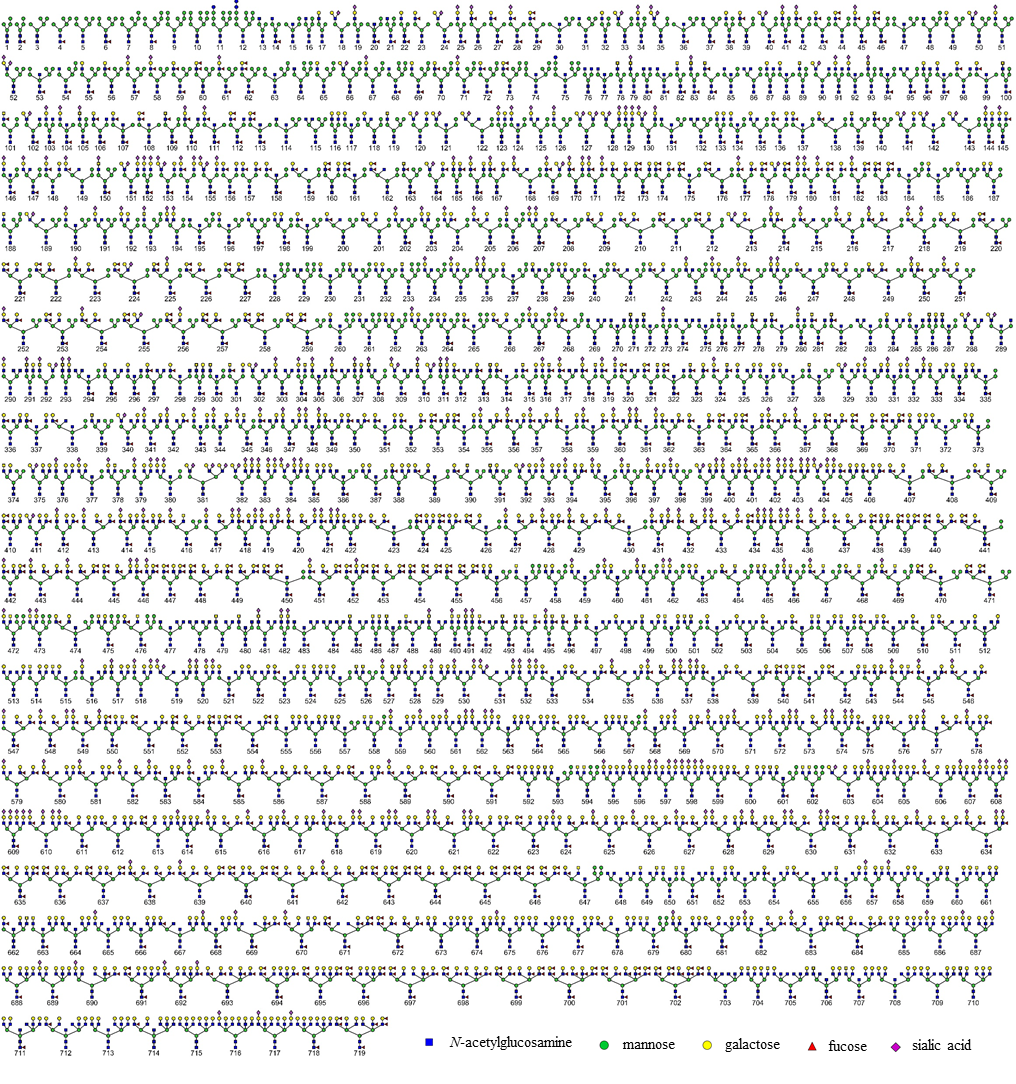
**

**Figure S1** Precise structures of 719 glycans identified in human spermatozoa.


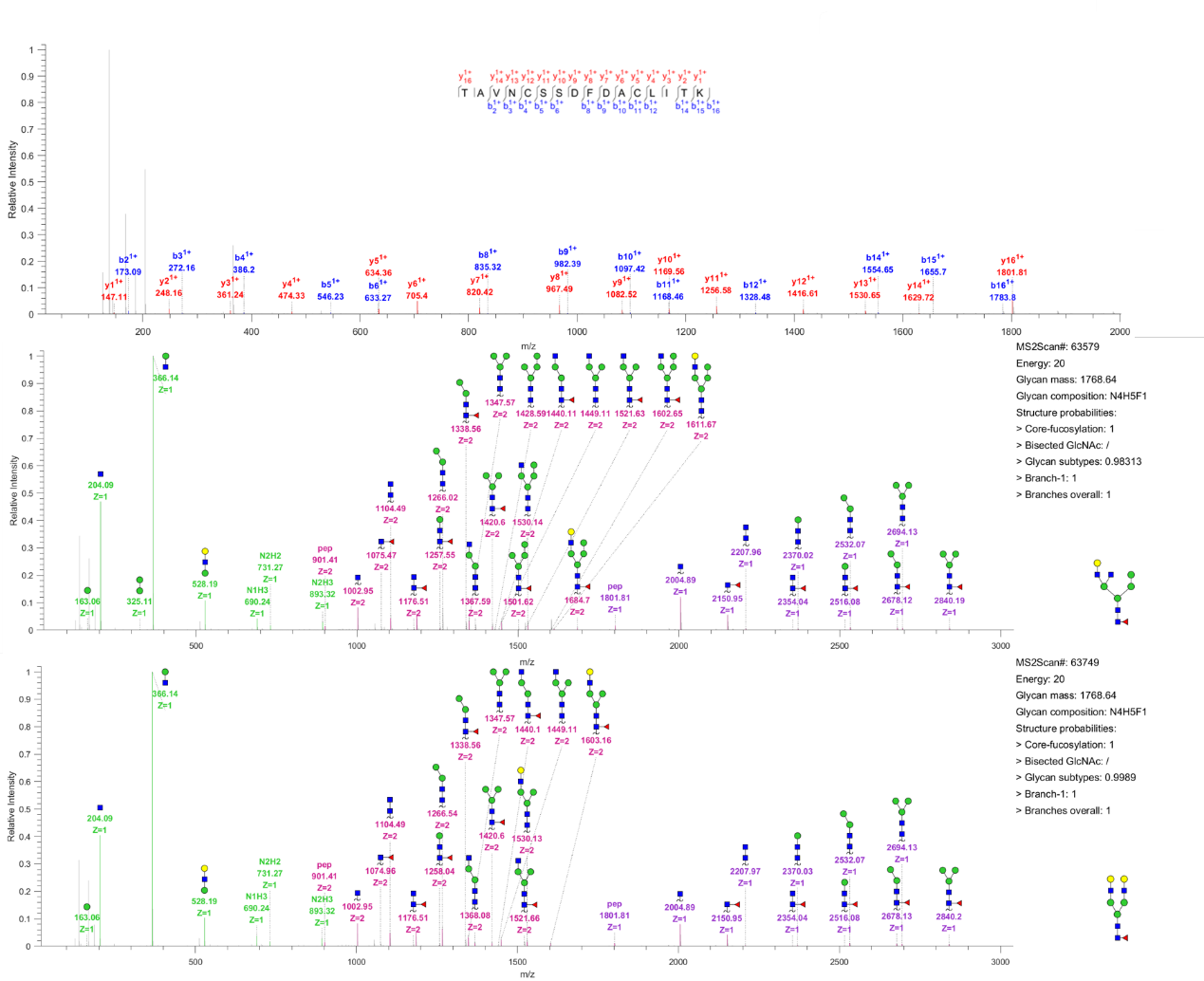


**Figure S2** Spectra of glycopeptides with two glycan isoforms of N4H5F1 on the same peptide. The peptide TAVNCSSDFDACLITK was modified by two different isoforms of the glycan HexNAc4Hex5Fuc1 (N4H5F1). Upper: peptide sequences identification using a MS/MS spectrum of high HCD energy (HCD = 33%). Lower: glycan structure determination using MS/MS spectra of low HCD energy (HCD = 20%).


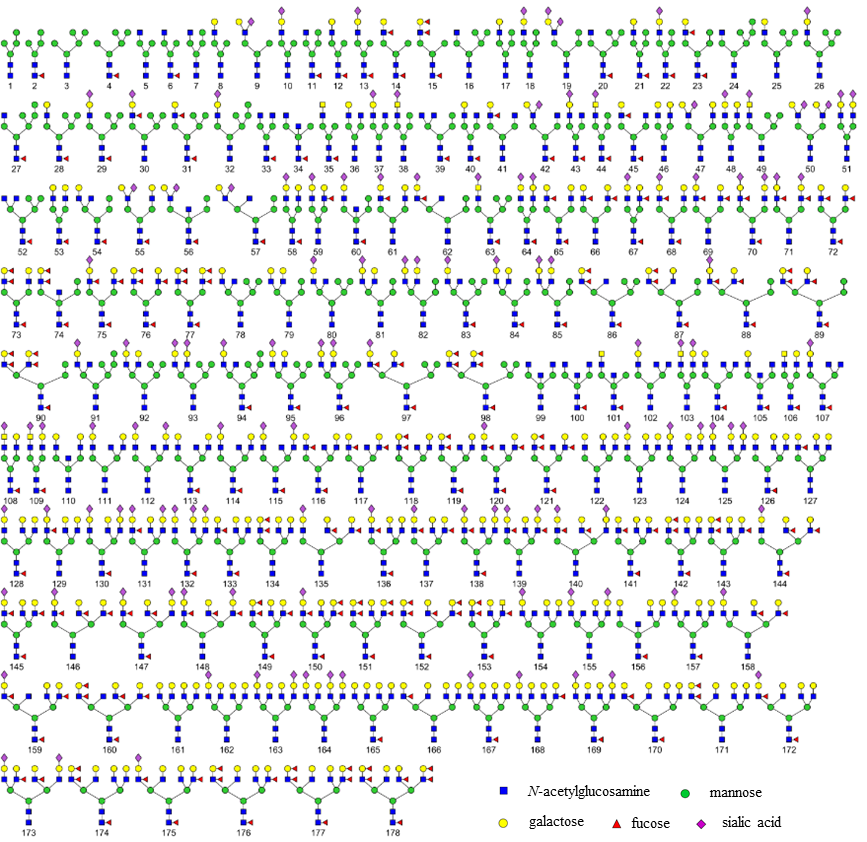


**Figure S3** The glycan structures on peptide: EIRHNSTGCLR of clusterin in human spermatozoa.


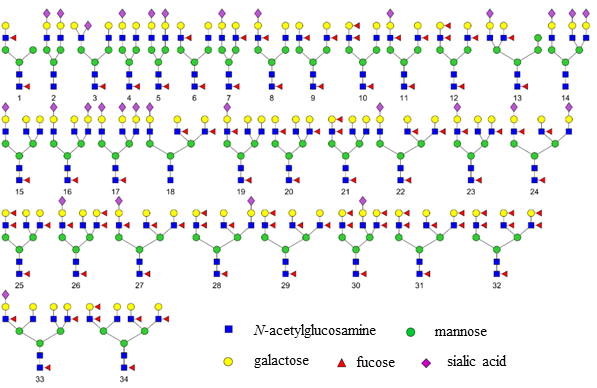


**Figure S4** The glycan structures on peptide: LKELPGVCNETMMALWEECKPCLK of clusterin in human spermatozoa.


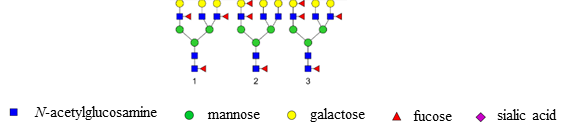


**Figure S5** The glycan structures on peptide: KKEDALNETR of clusterin in human spermatozoa.


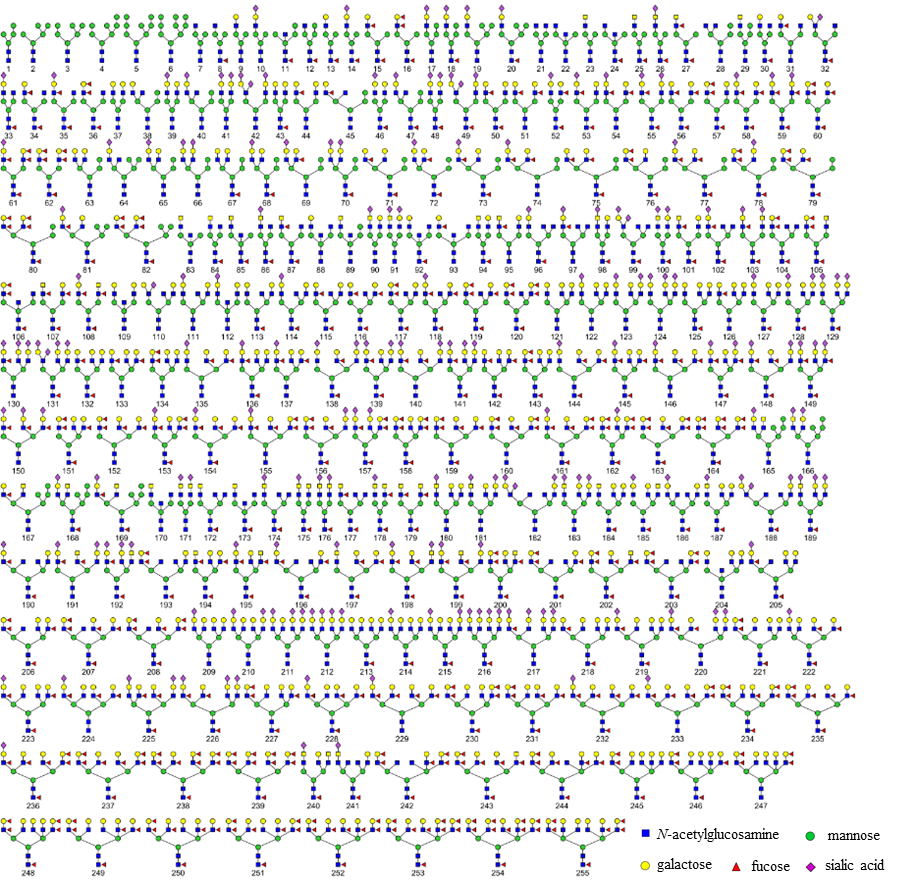


**Figure S6** The glycan structures on peptide: LANLTQGEDQYYLR of clusterin in human spermatozoa.


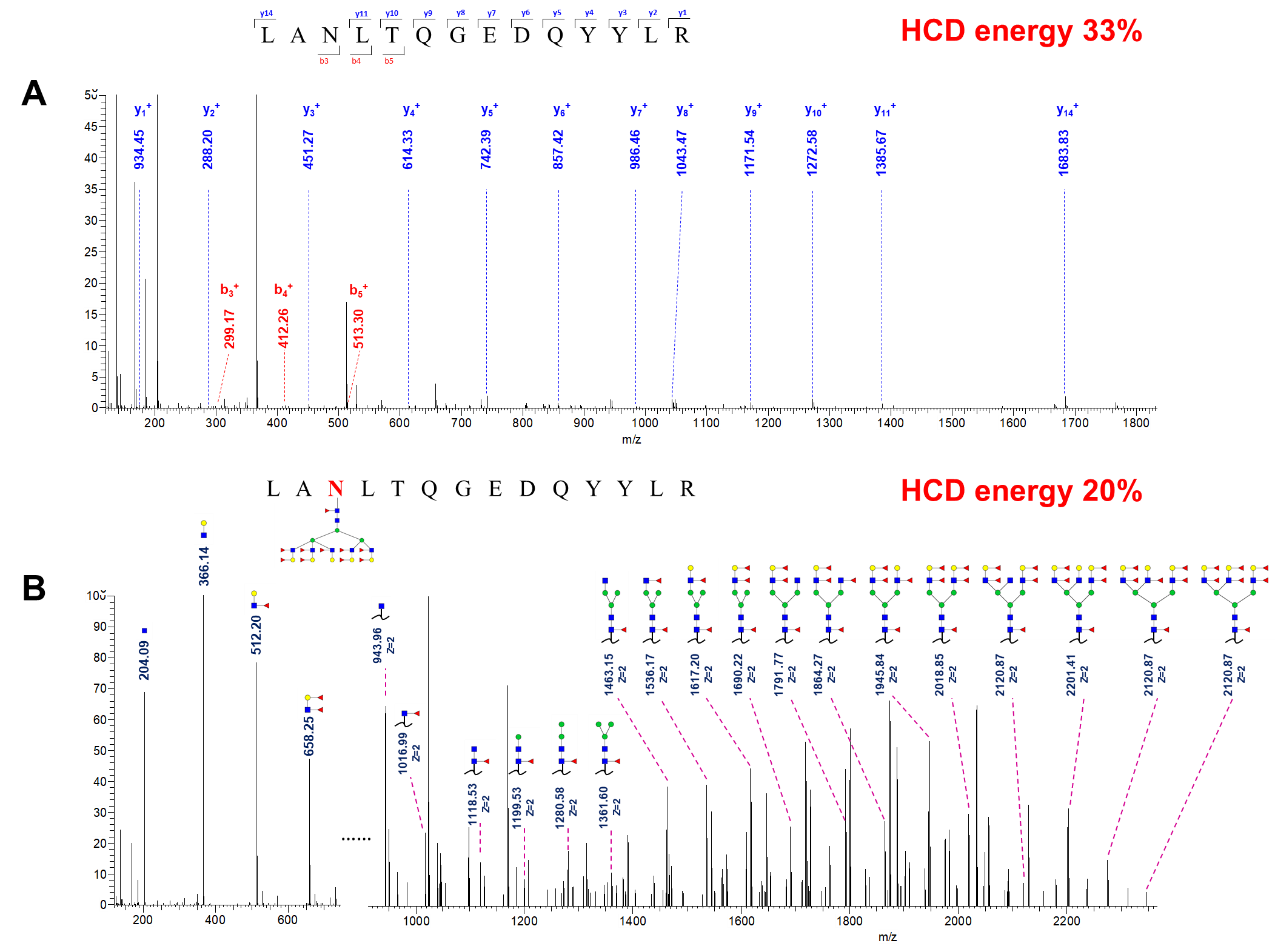


**Figure S7** The annotations of glycan structures N7H8F10 on clusterin in MS/MS data. (A) The peptide (peptide backbone: LANLTQGEDQYYLR) of a representative glycopeptide was identified at a high-energy HCD collision energy (33%). (B) The low-energy HCD (20%) spectrum containing B/Y ions of the glycan N7H8F10 from the intact glycopeptide. B ions include some special peaks such as N1H1F1, N1H1F2 which can be used to deduced a part of glycan structure. Y ions include large number of peaks which can be used to verify glycan structure.


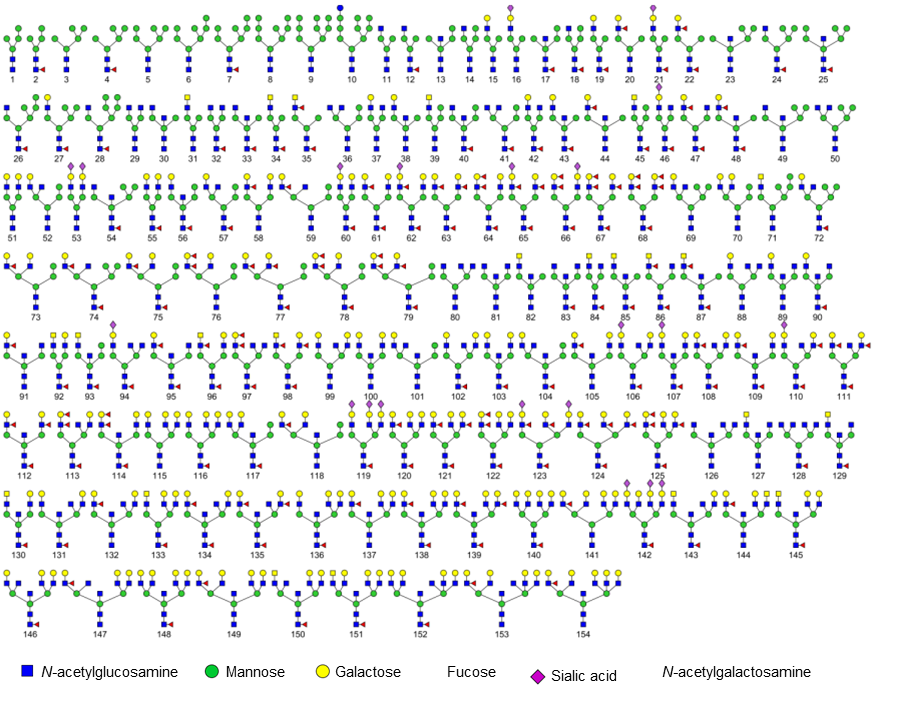


**Figure S8** The glycan structures on acrosome of human spermatozoa.

1. **Supplementary Tables**

**Table S1** Clinical information of semen parameters used in this study

| Code | Age | Volume  (mL) | Spermatozoa concentration (10⁶/ml) | Progressive motility rate (%) | Motility rate (%) | pH |
| --- | --- | --- | --- | --- | --- | --- |
| 1 | 27 | 4 | 69.6 | 47.1 | 55.6 | 7.2 |
| 2 | 28 | 3 | 116.1 | 42.8 | 44.8 | 7.2 |
| 3 | 29 | 6 | 75.4 | 41.5 | 50.2 | 7.2 |
| 4 | 30 | 3 | 53.4 | 39.7 | 42.2 | 7.2 |
| 5 | 30 | 5.3 | 38.6 | 51.9 | 60.9 | 7.6 |
| 6 | 32 | 4 | 40 | 39.3 | 41.7 | 7.2 |
| 7 | 32 | 3.1 | 80.4 | 53 | 59 | 7.6 |
| 8 | 33 | 4.5 | 49.5 | 60.4 | 61.8 | 7.2 |
| 9 | 33 | 3.9 | 110.4 | 58 | 64 | 7.5 |
| 10 | 34 | 4.9 | 56.5 | 68 | 73 | 7.7 |

**Table S4** Glycan compositions with different glycan isoforms identified in spermatozoa

| The number of glycan isoforms | Number of glycan compositions |
| --- | --- |
| 10 | 2 |
| 8 | 3 |
| 7 | 7 |
| 6 | 11 |
| 5 | 15 |
| 4 | 31 |
| 3 | 42 |
| 2 | 54 |
| 1 | 127 |
